# Recreational drug use and risks of HIV and sexually transmitted infections among Chinese men who have sex with men: Mediation through multiple sexual partnerships

**DOI:** 10.1186/s12879-014-0642-9

**Published:** 2014-12-02

**Authors:** Jun-Jie Xu, Chen Zhang, Qing-Hai Hu, Zhen-Xing Chu, Jing Zhang, Yong-Ze Li, Lin Lu, Zhe Wang, Ji-Hua Fu, Xi Chen, Hong-Jing Yan, Ming-Hua Zhuang, Yong-Jun Jiang, Wen-Qing Geng, Sten H Vermund, Hong Shang, Han-Zhu Qian

**Affiliations:** Key Laboratory of AIDS Immunology of National Health and Family Planning Commission, Department of The First Affiliated Hospital, China Medical University, 92 North Second Road, Shenyang, 110001 Heping District China; Collaborative Innovation Center for Diagnosis and Treatment of Infectious Diseases, Hangzhou, China; Vanderbilt Institute for Global Health and Departments of Pediatrics and Medicine, Vanderbilt University School of Medicine, 2525 West End Avenue, Suite 750, Nashville, 37203 TN USA; Yunnan Provincial Centers for Disease Control and Prevention (CDC), Kunming, China; Henan Provincial CDC, Zhengzhou, China; Shandong Provincial CDC, Jinan, China; Hunan Provincial CDC, Changsha, China; Jiangsu Provincial CDC, Nanjing, China; Shanghai Municipal CDC, Shanghai, China

**Keywords:** Men who have sex with men, Multiple sexual partners, Unprotected sex, HIV, Syphilis, HSV-2, Recreational drug use, Substance use, China

## Abstract

**Background:**

Recreational drug use (RDU) may result in sexual disinhibition and higher risk for HIV and other sexually transmitted infections (STIs) among men who have sex with men (MSM) in China. We assessed whether RDU was associated with HIV, syphilis, and herpes simplex virus type 2 (HSV-2) within the context of multiple sexual partnerships and unprotected sex.

**Methods:**

We conducted a cross-sectional study among sexually-active MSM in six Chinese cities (Kunming, Jinan, Changsha, Zhengzhou, Nanjing, and Shanghai) in 2012–2013. We interviewed participants regarding RDU and sexual activity and drew blood for HIV, syphilis, and HSV-2. We fit multiple logistic regression models to assess associations of drug use and HIV, syphilis and HSV-2 infections, controlling for number of sexual partners and unprotected sex.

**Results:**

Of 3830 participants, 28% reported ever using ≥1 of these drugs in the past 6 months: popper (alkyl nitrites), ecstasy, ice (methamphetamine), amphetamine, tramadol, and ketamine. In the past six months, 62% of MSM reported ≥2 sexual partners and 76% did not use condoms at last sexual encounter. HIV, syphilis and HSV-2 prevalences were 9.2%, 12.2%, and 10.3%, respectively.RDU was associated with HIV infection (aOR = 1.67; 95% CI, 1.31-2.13). Men with RDU were more likely to report multiple sexual partners (OR = 1.69; 95% CI, 1.44-1.98) and unprotected sex (aOR = 1.25; 95% CI, 1.05-1.49). The RDU-HIV association persisted (aOR = 1.58; 95% CI = 1.23-2.02) after adjusting for numbers of partners.

**Conclusions:**

RDU was associated with multiple sexual partnerships, unprotected sex, and HIV among Chinese MSM. It is plausible that RDU is a driver of increased sexual/HIV risk and/or may be an associated behavior with sexually risky lifestyles. Community engagement is needed.

**Electronic supplementary material:**

The online version of this article (doi:10.1186/s12879-014-0642-9) contains supplementary material, which is available to authorized users.

## Background

HIV risks in China have been shifting from injection drug use and contaminated plasma collection to unprotected sexual contacts in the last decade [1],[2], and the rapid increase of male-to-male HIV transmission is of particular concern [3]-[6]. From a tiny proportion of new HIV/AIDS cases in 2005, transmission among men who have sex with men (MSM) was reported for 70,000 (23%) of the new HIV cases in the first nine months of 2013 [4]-[7].

Unprotected anal intercourse is the primary risk factor for HIV infection among MSM [8],[9]. MSM who are recreational drug users may be more susceptible to infection of HIV and sexually transmitted infections (STIs) compared to their non-drug using peers [10]. A recent review showed a wide range (0.1% -44%; median 2.4%)of MSM reporting ever using recreational drugs, higher than that in the general population [1],[11]-[14].

Recreational drug use (RDU) may increase risk for HIV/STI acquisition by disinhibiting behavior, impairing judgment such that safer sex practices are bypassed, and/or increasing sexual desire [15]. Disinhibition and opting out of safer sex has been termed “cognitive escape” which undermines HIV prevention [15]-[17]. Most studies assess the relationship between RDU and sexual risk behavior [18], or examine HIV risk among drug users [10]. Some studies have shown sexual risk behaviors are associated with both RDU and HIV infection, suggesting that a more complex model should be employed to examine these associations. As expected, a mediating role of sexual risk behaviors in the pathway between RDU and acquisition of HIV and STIs has been observed [15]. We propose a conceptual model to examine the mediating role of sexual risk behaviors between RDU and risks of HIV, syphilis, and human simplex virus type 2 (HSV-2). We sought to examine further the associations of RDU, sexual risk, and HIV/STIs among MSM in urban China.

## Methods

### Study design and participants

We conducted a cross-sectional study in seven large Chinese cities including Kunming, Shenyang, Jinan, Changsha, Zhengzhou, Nanjing and Shanghai from June 2012 to June 2013. These cities represent different geographical locations, social and economic development, proximity to opiate drug use center, and HIV prevalence across China. For example, Kunming City is adjacent to the opiate drug epidemic center in the border regions of Yunnan Province in south-western China where HIV prevalence is high in the drug users and general population; Zhengzhou is the capital city of Henan Province in central China where a large number of rural farmers contracted HIV through unhygienic plasma collection two decades ago; Shanghai is the largest costal city in eastern China and has advanced social and economic development. As the data from Shenyang city have been published elsewhere [19], we only included participants from the rest six cities in the analysis. We recruited MSM participants using multiple approaches, including advertising on gay websites, outreach to gay-gathering venues (e.g., gay bars, parks, public bathhouses), and peer referral. Inclusion criteria were: male, 18 years or older, having lived in the study city for at least one year, self-reported ever having sex with other men, and providing informed consent.

### Data collection

A total of 3834 participants completed a questionnaire and undertook blood testing for HIV, syphilis, and HSV-2 infections. Participants took about 45 minutes to complete a questionnaire by themselves, and for those with low literacy (<5% of study sample), the questionnaire was administered by trained interviewer. The questionnaire collected the following information: (1) demographics, including age, marital status, ethnicity, education, and monthly income; (2) knowledge about HIV/AIDS; (3) HIV risk behaviors in the past six months, including number of sexual partners and condom use; and (4) recreational drug use in the past six months, including ever using the following types of drugs: popper (alkyl nitrites), ecstasy, ice (methamphetamine), amphetamine, tramadol, and ketamine. We also asked participants if they ever injected drugs in the past six months.

Laboratory testing of HIV, syphilis and HSV-2 was performed following the Chinese national standard protocols and laboratory methods (2010) [20]. For both HIV and HSV-2, initial screening was conducted using enzyme-linked immunosorbent assay (ELISA) method, and HIV positive cases were confirmed by Western blot (WB) test. A result was considered positive only if WB test was also positive. Rapid plasma reagin(RPR) and Treponemal Pallidum Particle Agglutination (TPPA) were used to diagnose syphilis and a result was considered positive only if both tests were positive. All laboratory tests were performed in AIDS research center of The First Affiliated Hospital of China Medical University, or the AIDS labs of each local Provincial CDC (Yunnan, Shandong, Hunan, Henan, Jiangsu and Shanghai).

After completing the questionnaire survey and giving a blood specimen, each participant received a small gift (e.g., umbrella, soap, and laundry detergent) with a cash value of about US$ 16.

### Data analysis

We dichotomized the primary predictor variable RDU as “1” if a participant used any drugs in the past 6 months, or“0” if he did not use any drugs. We created a new variable of multiple partnerships with a value of “1” if a participant reported ≥ 2 sexual partners in the past 6 months, or “0” if having only one or no sexual partner. We created a composite score for eight HIV/AIDS knowledge questions. For each question, the response was coded as a value of 1 for correct answer or 0 for incorrect answer. The composite score for HIV/AIDS knowledge was calculated by summing the number of correct answers, and a higher score indicates a higher level of HIV/AIDS knowledge.

We employed Chi-square (for categorical data) or t-test (for continuous data) to compare the difference in demographic, HIV knowledge and behavior variables and the prevalence of HIV, syphilis, and HSV-2 by participant’s drug use status. We performed eight series of regression models to test the mediation effect of multiple sexual partnerships on the association of RDU and risks of HIV, syphilis and HSV-2,using the methods of Baron and Kenny [21]. Multiple partnerships and unprotected sex served as mediators, drug use served as an independent variable, and HIV, syphilis or HSV-2infection served as separate dependent variables. For the mediation effect of multiple partnerships, we firstly regressed multiple partnerships on RDU, then regressed HIV, syphilis or HSV-2 infection on RDU and multiple partnerships, respectively. Finally, we regressed HIV, syphilis or HSV-2 on both RDU and multiple partnerships. Similarly, we tested the mediation effect of unprotected sex on the association between RDU and HIV, syphilis or HSV-2 infection. For each regression, age, marital status, monthly income, and HIV knowledge were controlled for, as these variables showed statistical significance in the bivariate analyses based upon their drug use status.

We estimated regression coefficients for each equation by adjusted odds ratios (aOR), 95% confidence intervals (CI). We conducted our data analyses using STATA® (12.0) for Windows (StataCorp®, College Station, Texas, USA), the STATA commands were shown in Additional file 1.

### Ethics issues

The study protocol was reviewed and approved by the ethics committee of The First Affiliated Hospital of China Medical University (2011{36}). Each reached MSM participant had the right to join or decline to attend this survey. Written informed consent for participation in the study was obtained from participants before this questionnaire survey.

## Results

### Demographics of study participants

Study-wide, 2.9% of MSM we approached refused to participate, with participation rates ranging from 94.5% to 98.5% in six study sites. A total of 3834 MSM completed the study. After exclusion of MSM who did not report their RDU status, 3830 participants were included in the final analysis. The mean age was 30 years, and the majority of participants were single (72%) and of Han ethnicity (95%), and had attended high school or college (82%).

### Recreational drug use, risky sex, and prevalence of HIV, syphilis and HSV-2

**Figure 1:**
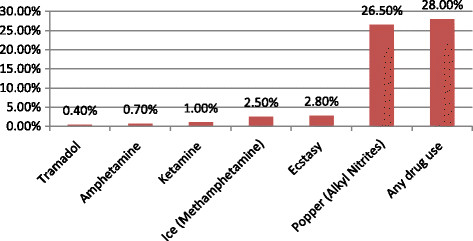
**Recreational drug use behaviour of China MSM.** Figure 1 showed the prevalence of self-reported recreational drug using behaviours in the past six months in MSM participants from six big cities of China (Kunming, Jinan, Changsha, Zhengzhou, Nanjing, and Shanghai) during 2012–2013. Twenty eight percent of the MSM participants used at least one kind of recreational drugs, and 26.5% used popper (Alkyl Nitrites).

Of 3,830 participants, 1,072 (28.0%) reported use of recreational drugs in the past six months, including popper (26.5%), ecstasy (2.8%), ice (2.5%), amphetamine (0.7%), tramadol (0.4%), and ketamine(1.0%) (Figure 1). In the past six months, 62.2% of participants reported multiple partners and 75.5% reported ever having unprotected sex. HIV, syphilis, and HSV-2 were diagnosed in 9.2%, 12.2% and 10.3% of men, respectively. Compared to non-users, drug users were significantly younger, had higher income, were more likely to be single, have multiple partnerships, and unprotected sex, and had lower knowledge scores and higher HIV prevalence(*P* < 0.05) (Table 1).

**Table 1:** **Basic characteristics and HIV/STIs prevalence among China MSM**

Variables	Total sample	Drug users	Non-drug users	*P* -value
	N(%)	N(%)	N(%)	
**Overall**	3830(100.0)	1072(28.0)	2758(72.0)	
**Education**				
*<High school*	689(18.0)	190(17.7)	499(18.1)	0.790
*≥High school*	3141(82.0)	882(82.3)	2259(81.9)	
**Marital status**				
*Ever married*	1061(27.7)	211(19.7)	850(31.0)	<0.01
*Never married*	2769(72.3)	861(80.3)	1908(69.2)	
**Ethnicity**				
*Han majority*	3638(95.0)	1020(95.2)	2618(94.9)	0.770
*Non-Han minorities*	192(5.0)	52(4.9)	140(5.1)	
**Personal monthly income (Yuan)**				
>3000	1603(41.9)	541(50.5)	1062(38.5)	<0.001
*≤3000*	2226(58.1)	530(49.5)	1696(61.5)	
**Age, year (mean, SD)**	30.5(9.5)	28.6(7.5)	31.3(10.2)	<0.001
**HIV knowledge (maximal score = 8) (mean, SD)**	7.4 (2.1)	6.7(2.3)	7.6(1.9)	<0.001
**Number of sexual partners in the past 6 months**				
*≤1*	1434(37.9)	308 (29.2)	1126(41.2)	<0.001
*≥2*	2355(62.2)	746 (70.8)	1609(58.8)	
**Unprotected sex in the last sex act**	2889(75.5)	839 (78.3)	2050(74.3)	0.010
**HIV infection**	354(9.2)	125 (11.7)	229(8.03)	<0.01
**Syphilis infection**	466(12.2)	157(14.7)	309(11.2)	<0.01
**HSV-2 infection**	394(10.3)	99(9.2)	295(10.7)	0.100

STIs: sexually transmitted infections; MSM: men who have sex with men; HSV-2: herpes simplex type 2; SD: standard deviation. *1 US dollar ≈ 6.25 Chinese yuan during conducting this study.

### Relationship between recreational drug use and HIV, syphilis and HSV-2 infections

RDU was positively associated with HIV infection (aOR, 1.67; 95% CI, 1.31-2.13), and syphilis (aOR, 1.61; 95% CI, 1.29-2.00) but not associated with HSV-2 (aOR, 0.96; 95% CI, 0.75-1.24). In the mediation analysis of treating multiple sexual partnerships as a potential mediator in the path of RDU and HIV infection, “multiple partnerships” was independently associated with both RDU (aOR, 1.69; 95% CI, 1.44-1.98) and HIV infection (aOR, 1.35; 95% CI, 1.06-1.71). When multiple partnerships and RDU were both included in the model, the association between RDU and HIV infection was confirmed (aOR, 1.58; 95% CI, 1.23-2.02), as was the association of multiple partnerships and HIV infection (aOR, 1.29; 95% CI, 1.02-1.64). Multiple partnerships served as mediator on the path of RDU and HIV infection (Table 2). However, no significant mediation effect was noted for syphilis and HSV-2 infections (Table 2).

**Table 2:** **The mediation effect of multiple sexual partners on recreational drug use and HIV/STIs infection of MSM**

Variables	X → M	M → Y	X → Y	(X, M) → Y
	aOR(95% CI) ^#^	aOR(95% CI) ^#^	aOR(95% CI) ^#^	aOR(95% CI) ^#^
**Outcome: HIV infection (Y)**				
*Drug use (X)*	1.69(1.44,1.98)**	N/A	1.67(1.31,2.13)**	1.58(1.23, 2.02)**
*Multiple partners (M)*	N/A	1.35(1.06,1.71)*	N/A	1.29(1.02,1.64)*
**Outcome: syphilis infection (Y)**				
*Drug use (X)*	1.69(1.44,1.98)**	1.24(1.01,1.52)*	1.61(1.29, 2.00)**	1.59(1.28,1.99)**
*Multiple partners (M)*	N/A	N/A	N/A	1.18(0.96,1.45)
**Outcome: HSV-2 infection (Y)**				
*Drug use (X)*	1.69(1.44,1.98)**	1.06(0,85,1.32)	0.96(0.75,1.24)	0.96(0.74, 1.23)
Multiple partners (M)	N/A	N/A	N/A	1.07(0.86,1.33)

Notes: MSM: men who have sex with men; HSV-2: herpes simplex type 2; N/A: not applicable; X: exposure variable; Y: outcome variable; M: mediator; aOR: adjusted odds ratio; CI: confidence interval.

^#^All multivariate regression models are controlled for the covariates which are significant in bivariate analyses in Table 1, including age, marital status, monthly income and HIV knowledge. *p < 0.05, **p < 0.01.

The analysis on the potential mediating role of unprotected sex on the path between RDU and HIV infection is presented in Table 3. As RDU was not significantly associated with HIV, syphilis or HSV-2, unprotected sex was unlikely to serve as a mediator in the relationship between RDU and these infections.

**Table 3:** **The mediation effect of unprotected anal sex on recreational drug use and HIV/STIs infection of MSM**

Variables	X → M	M → Y	X → Y	(X, M) → Y
	aOR(95% CI) ^#^	aOR(95% CI) ^#^	aOR(95% CI) ^#^	aOR(95% CI) ^#^
**Outcome: HIV infection (Y)**				
*Drug use (X)*	1.25(1.05,1.49)*	N/A	1.67(1.31,2.13)	1.69(1.32, 2.15)**
*Unprotected sex (M)*	N/A	0.81(0.63,1.03)	N/A	0.77(0.61,0.99)*
**Outcome: syphilis infection (Y)**				
*Drug use (X)*	1.25(1.05,1.49)*	N/A	1.61(1.29, 2.00)**	1.62(1.30, 2.02)**
*Unprotected sex (M)*	N/A	0.84(0,67,1.04)	N/A	0.82(0.65,1.02)
**Outcome: HSV-2 infection (Y)**				
*Drug use (X)*	1.25(1.05,1.49)*	N/A	0.96(0.75,1.24)	0.96(0.75,1.24)
*Unprotected sex (M)*	N/A	0.99(0.77,1.26)	N/A	0.98(0.77, 1.25)

Notes: STIs: sexually transmitted infections; MSM: men who have sex with men; HSV-2: herpes simplex type 2; N/A: not applicable; X: exposure variable;, Y: outcome variable;, M: mediator.; aOR: adjusted odds ratio; CI: confidence interval.

^#^All multivariate regression models are controlled for the covariates which are significant in bivariate analyses in Table 1, including age, marital status, monthly income and HIV knowledge. *p < 0.05, **p < 0.01.

## Discussion

HIV was more common in MSM with multiple partners and using recreational drugs. A non-significant trend was seen for RDU and syphilis, but no association was noted for HSV-2. The mediation models suggested a strong role of RDU and multiple partnerships for predicting HIV risk, with less influence from unprotected sex. Prevention programs for Chinese MSM are not heavily dependent on condom promotion; RDU and partner numbers are foci for further community engagement and education. We cannot say whether RDU is a contributor to higher risk behaviors, or is a part of a matrix of linked activities, as when high risk sex is pre-planned along with RDU. While we found only 0.42% of participants reported having ever injected drugs in the past six months, use of non-injected recreational drugs was common among Chinese MSM [1] with 28.5% of participants reporting their use in the past six months. We believe that RDU is a neglected topic in current MSM risk reduction interventions in China [22]-[25].

The link between RDU and HIV risk has been described among MSM populations worldwide [19],[26]-[28], and RDU has also been linked to increased risk behaviors in MSM [29],[30]. However, there is sparse empiric evidence about the potential role of risky sex in the path between RDU and HIV infection in China. Our mediation analysis suggested that multiple partnerships, but not unprotected sex, acted as a mediator in the relationship between RDU and HIV infection. Since condom use clearly diminishes HIV risk, we speculate that our limited self-reported measure of condom use in the current study did not capture the effect of unprotected sex; we only asked participants if they used condom in their last sex act, and this did not measure long-term effects of unprotected sex in the acquisition of HIV and other STIs. Alternatively, condom use may not be a sensitive indicator of HIV risk among MSM [5].

The strengths of this study included a large sample size from multiple large Chinese cities, and using definitive laboratory tests with confirmations to measure HIV, syphilis and HSV-2 infections. Our mediation analysis helps advance the causal models. Limitations of the study include the limits of a cross-sectional study design that do not permit causal inferences between RDU and HIV, syphilis, and HSV-2 infections. A second limitation is that homosexuality in China is illegal and stigmatized, such that our data regarding sexual behaviors may be subject to socially desirable bias. Third, there are likely other unmeasured mediators on the pathways between drug use and HIV infection. Last, though the six large study cities represent different geographical locations and HIV prevalence in general population, the survey employed a convenience sample of study cities, and participants from these cities may not represent all Chinese MSM, as those who live in small cities and towns may have different drug use and sexual behaviors.

## Conclusions

This study extends existing literature by examining the mediating role of sexual risk behaviors on the path between drug use and infections of HIV, syphilis and HSV-2. To address the issue of increased risk sex among drug using MSM, HIV intervention programs may use innovative approaches, like “behavioral-structural” approach which combines behavioral, psychological and biomedical components to curtail these ongoing epidemics [31],[32]. First, HIV interventions among MSM should not only screen drug use, but also reduce sexual risk behaviors. Second, although the Chinese government has launched multiple large harm reduction programs, e.g., methadone maintenance treatment [33], these programs target persons who inject drugs, and programs for MSM are limited [1]. MSM face double barriers for access to harm reduction programs, including illicit nature of drug use and stigma attached to homosexuality. Chinese harm reduction programs should address RDU and make drug treatment services more widely accessible to MSM population. Third, due to the prevailing homophobia in China, MSM are usually hidden and invisible from health professionals and policy makers. Peer-led risk reduction interventions led by gay-friendly community-based organizations may be more effective among Chinese MSM than formal government health sector programs [34]. Fourth, interventions employing longer follow-up time to help promote behavior self-management skills are more likely to be effective for MSM risk reduction than more superficial engagements [35].MSM in China represent the fastest growing group of persons at risk and RDU is a neglected topic vis-à-vis risk in this key population.

## Additional file

## Electronic supplementary material

Additional file 1: Stata codes for the data analysis between recreational drug use and risks of HIV/STIs among Chinese MSM. (ZIP 8 KB)

Below are the links to the authors’ original submitted files for images.

Authors’ original file for figure 1
