# Supplementary figures and images for: Recreational drug use and risks of HIV and sexually transmitted infections among Chinese men who have sex with men: Mediation through multiple sexual partnerships
Source: BMC Infect Dis. 2014 Dec 2;14:642. doi: 10.1186/s12879-014-0642-9 (PMC4272794; doi:10.1186/s12879-014-0642-9)

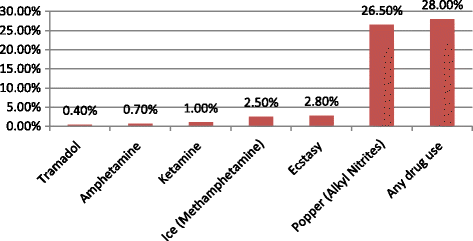

Supplement: Supplementary file 2 — Authors’ original file for figure 1 [file 12879_2014_642_MOESM2_ESM.gif]
